# Supplementary material for: Comparing the effects of climate change labelling on reactions of the Taiwanese public
Source: Nat Commun. 2020 Nov 27;11:6052. doi: 10.1038/s41467-020-19979-0 (PMC7699618; doi:10.1038/s41467-020-19979-0)
Supplement: Supplementary file 1 — Supplementary Information [file 41467_2020_19979_MOESM1_ESM.pdf]

Supplementary Information

Comparing the effects of climate change labelling on reactions of the Taiwanese public

Hung et al

Supplementary Table 1 Two-sided independent sample *t* test results of labelling effects for the subgroups – gender

Construct	Label	N	Mean(s.d.)	P values	Effect size (r)
Gender: male					
Communication – discuss with family members					
	Climate change	456	2.31(1.08)	.023	.08
	Climate crisis	440	2.15(1.01)		
Communication – discuss with friends					
	Climate change	462	2.5(.98)	.008	.09
	Climate crisis	444	2.32(1.02)		
Communications – information received					
	Climate change	449	2.88(1.01)	.002	.1
	Climate crisis	432	2.67(1.04)		
Belief – personal harm					
	Climate change	434	3.08(1.19)	.78	.01
	Climate crisis	414	3.06(1.17)		
Belief – harm future generations					
	Climate change	436	4.01(.91)	.636	.02
	Climate crisis	414	3.98(.99)		
Belief – worry					
	Climate change	456	3.97(1.04)	.426	.03
	Climate crisis	437	3.91(1.08)		
Belief – personal importance					
	Climate change	455	4.32(.85)	.739	.01
	Climate crisis	441	4.3(.89)		
Behavioural intention					
	Climate change	449	4.28(.87)	.168	.05
	Climate crisis	436	4.2(.92)		
Involvement – priority for governments					
	Climate change	442	2.91(.84)	.233	.04
	Climate crisis	416	2.84(.89)		
Belief – personal moral					

obligations					
	Climate change	455	4.2(1.02)	.878	.01
	Climate crisis	433	4.21(.94)		
Belief – moral obligations for future generations					
	Climate change	457	4.41(.84)	.136	.05
	Climate crisis	434	4.32(.89)		
Belief – collective efficacy					
	Climate change	445	4.29(.97)	.177	.05
	Climate crisis	430	4.2(1.05)		
Involvements – climate issue important for voting decision					
	Climate change	429	3.38(1.42)	.47	.02
	Climate crisis	412	3.31(1.46)		
Gender: female					
Communication – discuss with family members					
	Climate change	467	2.49(1.08)	.69	.01
	Climate crisis	507	2.47(1.05)		
Communication – discuss with friends					
	Climate change	470	2.5(1.04)	.932	0
	Climate crisis	510	2.49(.98)		
Communications – information received					
	Climate change	465	2.95(1.07)	.837	.01
	Climate crisis	505	2.96(1.07)		
Belief – personal harm					
	Climate change	440	3.22(1.13)	.078	.06
	Climate crisis	484	3.35(1.06)		
Belief – harm future generations					
	Climate change	449	4.04(.94)	.215	.04
	Climate crisis	487	4.12(.84)		
Belief – worry					
	Climate change	463	4.13(.95)	.119	.05
	Climate crisis	503	4.21(.78)		
Belief – personal importance					

	Climate change	462	4.4(.83)	.633	.02
	Climate crisis	504	4.42(.74)		
Behavioural intention					
	Climate change	455	4.31(.78)	.018	.08
	Climate crisis	499	4.42(.66)		
Involvement – priority for governments					
	Climate change	442	2.95(.81)	.438	.03
	Climate crisis	484	2.91(.81)		
Belief – personal moral obligations					
	Climate change	459	4.29(.93)	.106	.05
	Climate crisis	497	4.38(.79)		
Belief – moral obligations for future generations					
	Climate change	462	4.4(.85)	.074	.06
	Climate crisis	502	4.49(.69)		
Belief – collective efficacy					
	Climate change	444	4.28(.95)	.274	.04
	Climate crisis	493	4.34(.89)		
Involvements – climate issue important for voting decision					
	Climate change	441	3.3(1.41)	.314	.03
	Climate crisis	479	3.39(1.32)		

Supplementary Table 2 Two-sided independent sample *t* test results of labelling effects for the subgroups – age

Construct	Label	N	Mean(s.d.)	P values	Effect size (r)
Age: 20-49 years-old					
Communication – discuss with family members					
	Climate change	438	2.43(1.01)	.378	.03
	Climate crisis	424	2.37(1.05)		
Communication – discuss with friends					
	Climate change	439	2.52(.9)	.159	.05
	Climate crisis	424	2.43(.97)		
Communications – information received					
	Climate change	439	2.98(.96)	.467	.02
	Climate crisis	419	2.93(1)		
Belief – personal harm					
	Climate change	428	3.17(1.06)	.27	.04
	Climate crisis	416	3.25(1.03)		
Belief – harm future generations					
	Climate change	432	4.06(.86)	.452	.03
	Climate crisis	418	4.11(.88)		
Belief – worry					
	Climate change	437	4.02(.95)	.673	.01
	Climate crisis	421	4.04(.9)		
Belief – personal importance					
	Climate change	437	4.28(.86)	.939	0
	Climate crisis	423	4.28(.86)		
Behavioural intention					
	Climate change	435	4.23(.83)	.932	0
	Climate crisis	422	4.23(.82)		
Involvement – priority for governments					
	Climate change	435	2.85(.77)	.167	.05
	Climate crisis	419	2.78(.81)		
Belief – personal moral					

obligations					
	Climate change	439	4.29(.94)	.817	.01
	Climate crisis	422	4.3(.83)		
Belief – moral obligations for future generations					
	Climate change	438	4.41(.79)	.434	.03
	Climate crisis	422	4.37(.75)		
Belief – collective efficacy					
	Climate change	435	4.25(.93)	.911	0
	Climate crisis	419	4.24(.98)		
Involvements – climate issue important for voting decision					
	Climate change	429	3.16(1.39)	.717	.01
	Climate crisis	412	3.12(1.4)		
Age: 50 years-old and above					
Communication – discuss with family members					
	Climate change	482	2.38(1.15)	.185	.04
	Climate crisis	519	2.28(1.1)		
Communication – discuss with friends					
	Climate change	490	2.47(1.09)	.242	.04
	Climate crisis	526	2.39(1.02)		
Communications – information received					
	Climate change	472	2.85(1.11)	.1	.05
	Climate crisis	514	2.74(1.11)		
Belief – personal harm					
	Climate change	443	3.13(1.26)	.481	.02
	Climate crisis	480	3.18(1.19)		
Belief – harm future generations					
	Climate change	450	3.99(.98)	784	.01
	Climate crisis	481	4.01(.95)		
Belief – worry					
	Climate change	479	4.07(1.04)	.729	.01
	Climate crisis	515	4.1(.97)		
Belief – personal importance					

	Climate change	477	4.43(.81)	.948	0
	Climate crisis	518	4.43(.78)		
Behavioural intention					
	Climate change	467	4.36(.82)	.493	.02
	Climate crisis	509	4.39(.78)		
Involvement – priority for governments					
	Climate change	446	3.01(.86)	.39	.03
	Climate crisis	478	2.96(.87)		
Belief – personal moral obligations					
	Climate change	472	4.2(1.02)	.112	.05
	Climate crisis	505	4.3(.91)		
Belief – moral obligations for future generations					
	Climate change	478	4.39(.89)	372	.03
	Climate crisis	511	4.44(.83)		
Belief – collective efficacy					
	Climate change	451	4.31(.99)	.913	0
	Climate crisis	500	4.31(.95)		
Involvements – climate issue important for voting decision					
	Climate change	438	3.51(1.42)	.627	.02
	Climate crisis	477	3.55(1.35)		

Supplementary Table 3 Two-sided independent sample *t* test results of labelling effects for the subgroups – educational attainment

Construct	Label	N	Mean(s.d.)	P values	Effect size (r)
Educational attainment: high school or below					
Communication – discuss with family members					
	Climate change	399	2.2(1.1)	.076	.06
	Climate crisis	415	2.07(1.07)		
Communication – discuss with friends					
	Climate change	406	2.32(1.06)	.119	.05
	Climate crisis	421	2.2(1.03)		
Communications – information received					
	Climate change	390	2.63(1.13)	.324	.03
	Climate crisis	407	2.55(1.1)		
Belief – personal harm					
	Climate change	372	3.02(1.28)	.4	.03
	Climate crisis	378	3.09(1.21)		
Belief – harm future generations					
	Climate change	376	3.9(1.04)	.774	.01
	Climate crisis	376	3.92(.99)		
Belief – worry					
	Climate change	394	3.96(1.08)	.629	.02
	Climate crisis	408	3.99(1.02)		
Belief – personal importance					
	Climate change	393	4.38(.86)	.613	.02
	Climate crisis	413	4.35(.84)		
Behavioural intention					
	Climate change	384	4.25(.88)	.432	.03
	Climate crisis	403	4.3(.82)		
Involvement – priority for governments					
	Climate change	366	2.93(.87)	.924	0
	Climate crisis	376	2.92(.87)		

Belief – personal moral obligations					
	Climate change	391	4.18(1.05)	.847	.01
	Climate crisis	399	4.2(.98)		
Belief – moral obligations for future generations					
	Climate change	395	4.35(.95)	.646	.02
	Climate crisis	404	4.38(.86)		
Belief – collective efficacy					
	Climate change	374	4.23(1.06)	.932	0
	Climate crisis	398	4.23(1.02)		
Involvements – climate issue important for voting decision					
	Climate change	357	3.43(1.43)	.697	.01
	Climate crisis	375	3.47(1.39)		
Educational attainment: some college and above					
Communication – discuss with family members					
	Climate change	523	2.56(1.04)	.516	.02
	Climate crisis	529	2.52(.97)		
Communication – discuss with friends					
	Climate change	525	2.63(.94)	.299	.03
	Climate crisis	530	2.57(.95)		
Communications – information received					
	Climate change	523	3.13(.91)	.118	.05
	Climate crisis	527	3.04(.99)		
Belief – personal harm					
	Climate change	501	3.25(1.06)	.482	.02
	Climate crisis	517	3.3(1.04)		
Belief – harm future generations					
	Climate change	509	4.12(.82)	.57	.02
	Climate crisis	522	4.15(.85)		
Belief – worry					
	Climate change	524	4.12(.93)	.722	.01

	Climate crisis	529	4.14(.87)		
Belief – personal importance					
	Climate change	523	4.35(.82)	.475	.02
	Climate crisis	529	4.38(.8)		
Behavioural intention					
	Climate change	519	4.33(.79)	.99	0
	Climate crisis	529	4.33(.79)		
Involvement – priority for governments					
	Climate change	518	2.93(.79)	.088	.05
	Climate crisis	521	2.85(.83)		
Belief – personal moral obligations					
	Climate change	522	4.29(.92)	.098	.05
	Climate crisis	529	4.38(.77)		
Belief – moral obligations for future generations					
	Climate change	523	4.45(.75)	.816	.01
	Climate crisis	529	4.44(.75)		
Belief – collective efficacy					
	Climate change	514	4.32(.88)	.744	.01
	Climate crisis	522	4.3(.93)		
Involvements – climate issue important for voting decision					
	Climate change	512	3.27(1.4)	.914	0
	Climate crisis	515	3.26(1.39)		

Supplementary Table 4 Two-sided independent sample *t* test results of labelling effects for the subgroups – cultural worldviews

Construct	Label	N	Mean(s.d.)	P values	Effect size (r)
People with equalitarian worldviews					
Communication – discuss with family members					
	Climate change	387	2.48(1.13)	.628	.02
	Climate crisis	409	2.44(1.08)		
Communication – discuss with friends					
	Climate change	391	2.59(1)	.638	.02
	Climate crisis	411	2.56(1.03)		
Communications – information received					
	Climate change	388	2.99(1.03)	.381	.03
	Climate crisis	407	2.93(1.1)		
Belief – personal harm					
	Climate change	373	3.34(1.15)	.545	.02
	Climate crisis	398	3.39(1.09)		
Belief – harm future generations					
	Climate change	383	4.17(.88)	.433	.03
	Climate crisis	401	4.22(.89)		
Belief – worry					
	Climate change	388	4.26(.89)	.509	.02
	Climate crisis	408	4.21(.9)		
Belief – personal importance					
	Climate change	387	4.52(.71)	.358	.03
	Climate crisis	411	4.47(.74)		
Behavioural intention					
	Climate change	384	4.37(.77)	.469	.03
	Climate crisis	406	4.41(.74)		
Involvement – priority for governments					
	Climate change	379	3.12(.77)	.13	.05
	Climate crisis	396	3.03(.85)		

Belief – personal moral obligations					
	Climate change	388	4.33(.95)	.171	.05
	Climate crisis	403	4.41(.77)		
Belief – moral obligations for future generations					
	Climate change	391	4.49(.78)	.464	.03
	Climate crisis	406	4.52(.71)		
Belief – collective efficacy					
	Climate change	382	4.32(.95)	.059	.07
	Climate crisis	405	4.44(.87)		
Involvements – climate issue important for voting decision					
	Climate change	371	3.44(1.42)	.608	.02
	Climate crisis	391	3.49(1.35)		
People with hierarchical worldviews					
Communication – discuss with family members					
	Climate change	441	2.43(1.02)	.117	.05
	Climate crisis	443	2.33(.98)		
Communication – discuss with friends					
	Climate change	443	2.49(.99)	.464	.02
	Climate crisis	445	2.44(.93)		
Communications – information received					
	Climate change	438	2.97(.99)	.095	.06
	Climate crisis	438	2.86(.99)		
Belief – personal harm					
	Climate change	423	3.09(1.11)	.557	.02
	Climate crisis	420	3.14(1.04)		
Belief – harm future generations					
	Climate change	427	4.01(.85)	.517	.02
	Climate crisis	429	3.97(.84)		
Belief – worry					
	Climate change	440	3.98(.97)	.24	.04

	Climate crisis	440	4.06(.86)		
Belief – personal importance					
	Climate change	439	4.32(.81)	.714	.01
	Climate crisis	443	4.34(.78)		
Behavioural intention					
	Climate change	437	4.32(.78)	.968	0
	Climate crisis	440	4.31(.79)		
Involvement – priority for governments					
	Climate change	435	2.84(.82)	.225	.04
	Climate crisis	429	2.77(.82)		
Belief – personal moral obligations					
	Climate change	439	4.28(.91)	.893	0
	Climate crisis	441	4.27(.87)		
Belief – moral obligations for future generations					
	Climate change	440	4.44(.79)	.392	.03
	Climate crisis	442	4.39(.77)		
Belief – collective efficacy					
	Climate change	427	4.33(.9)	.02	.08
	Climate crisis	434	4.18(.99)		
Involvements – climate issue important for voting decision					
	Climate change	428	3.32(1.41)	.824	.01
	Climate crisis	425	3.29(1.39)		
People with individualistic worldviews					
Communication – discuss with family members					
	Climate change	23	2.65(1.07)	.007	.36
	Climate crisis	33	1.88(.96)		
Communication – discuss with friends					
	Climate change	23	2.35(1.03)	.009	.34
	Climate crisis	34	1.68(.84)		
Communications – information received					

	Climate change	22	2.59(1.22)	.098	.22
	Climate crisis	34	2.12(.88)		
Belief – personal harm					
	Climate change	22	2.41(1.33)	.724	.05
	Climate crisis	31	2.55(1.46)		
Belief – harm future generations					
	Climate change	19	3.21(1.36)	.798	.04
	Climate crisis	29	3.31(1.28)		
Belief – worry					
	Climate change	22	3.5(1.34)	.546	.08
	Climate crisis	33	3.27(1.38)		
Belief – personal importance					
	Climate change	21	3.9(1.18)	.772	.04
	Climate crisis	35	3.8(1.37)		
Behavioural intention					
	Climate change	22	4.05(1.09)	.562	.08
	Climate crisis	34	3.88(.98)		
Involvement – priority for governments					
	Climate change	21	2.29(.85)	.229	.17
	Climate crisis	30	2.63(1.1)		
Belief – personal moral obligations					
	Climate change	22	3.77(1.31)	.834	.03
	Climate crisis	33	3.85(1.3)		
Belief – moral obligations for future generations					
	Climate change	23	4(1.04)	.597	.07
	Climate crisis	34	3.82(1.34)		
Belief – collective efficacy					
	Climate change	21	4.33(.73)	.107	.22
	Climate crisis	34	3.85(1.21)		
Involvements – climate issue important for voting decision					
	Climate change	20	3.35(1.46)	.326	.14
	Climate crisis	32	2.91(1.63)		
People with fatalism					

worldviews					
Communication – discuss with family members					
	Climate change	48	1.85(.92)	.937	.01
	Climate crisis	37	1.84(.96)		
Communication – discuss with friends					
	Climate change	48	2.06(1.02)	.129	.17
	Climate crisis	38	1.74(.92)		
Communications – information received					
	Climate change	47	2.13(1.08)	.132	.17
	Climate crisis	37	2.49(1.07)		
Belief – personal harm					
	Climate change	41	2.68(1.25)	.428	.09
	Climate crisis	35	2.91(1.27)		
Belief – harm future generations					
	Climate change	37	3.49(1.04)	.279	.13
	Climate crisis	34	3.76(1.1)		
Belief – worry					
	Climate change	45	3.38(1.21)	.03	.24
	Climate crisis	36	3.94(1.09)		
Belief – personal importance					
	Climate change	45	3.84(1.19)	.121	.17
	Climate crisis	35	4.23(.94)		
Behavioural intention					
	Climate change	43	3.77(1.09)	.332	.11
	Climate crisis	36	4(1.01)		
Involvement – priority for governments					
	Climate change	38	2.55(.86)	.293	.12
	Climate crisis	36	2.75(.73)		
Belief – personal moral obligations					
	Climate change	45	3.76(1.15)	.275	.12
	Climate crisis	38	4.03(1.08)		
Belief – moral obligations for					

future generations					
	Climate change	46	3.98(1.02)	.224	.13
	Climate crisis	37	4.24(.93)		
Belief – collective efficacy					
	Climate change	42	3.74(1.19)	.133	.17
	Climate crisis	38	4.13(1.12)		
Involvements – climate issue important for voting decision					
	Climate change	40	2.63(1.25)	.156	.17
	Climate crisis	34	3.06(1.35)		
