# Supplementary material for: Comparing the effects of climate change labelling on reactions of the Taiwanese public
Source: Nat Commun. 2020 Nov 27;11:6052. doi: 10.1038/s41467-020-19979-0 (PMC7699618; doi:10.1038/s41467-020-19979-0)
Supplement: Supplementary file 3 — Reporting Summary [file 41467_2020_19979_MOESM3_ESM.pdf]

nature research

Corresponding author(s): Li-San Hung

Last updated by author(s): August 26, 2020

Reporting Summary

Nature Research wishes to improve the reproducibility of the work that we publish. This form provides structure for consistency and transparency in reporting. For further information on Nature Research policies, see our Editorial Policies and the Editorial Policy Checklist.

Statistics

For all statistical analyses, confirm that the following items are present in the figure legend, table legend, main text, or Methods section.

n/a	Confirmed	
☐	☒	The exact sample size (n) for each experimental group/condition, given as a discrete number and unit of measurement
☐	☒	A statement on whether measurements were taken from distinct samples or whether the same sample was measured repeatedly
☐	☒	The statistical test(s) used AND whether they are one- or two-sided<br>*Only common tests should be described solely by name; describe more complex techniques in the Methods section.*
☐	☒	A description of all covariates tested
☐	☒	A description of any assumptions or corrections, such as tests of normality and adjustment for multiple comparisons
☐	☒	A full description of the statistical parameters including central tendency (e.g. means) or other basic estimates (e.g. regression coefficient) AND variation (e.g. standard deviation) or associated estimates of uncertainty (e.g. confidence intervals)
☐	☒	For null hypothesis testing, the test statistic (e.g. F, t, r) with confidence intervals, effect sizes, degrees of freedom and P value noted<br>*Give P values as exact values whenever suitable.*
☒	☐	For Bayesian analysis, information on the choice of priors and Markov chain Monte Carlo settings
☒	☐	For hierarchical and complex designs, identification of the appropriate level for tests and full reporting of outcomes
☐	☒	Estimates of effect sizes (e.g. Cohen's d, Pearson's r), indicating how they were calculated

Our web collection on statistics for biologists contains articles on many of the points above.

Software and code

Policy information about availability of computer code

Data collection	Data was collected using Computer Assisted Telephone Interview (CATI) system.
Data analysis	The two-sided independent-samples t tests and Mann-Whitney Tests were performed in SPSS version 23 (Microsoft Windows version). The effect sizes for two-sided independent-samples t tests and Mann-Whitney Tests were calculated in Microsoft Excel version 2016.

For manuscripts utilizing custom algorithms or software that are central to the research but not yet described in published literature, software must be made available to editors and reviewers. We strongly encourage code deposition in a community repository (e.g. GitHub). See the Nature Research guidelines for submitting code & software for further information.

Data

Policy information about availability of data

All manuscripts must include a data availability statement. This statement should provide the following information, where applicable:
- Accession codes, unique identifiers, or web links for publicly available datasets
- A list of figures that have associated raw data
- A description of any restrictions on data availability

The data that support the findings of this study are available from the corresponding author upon reasonable request.

Field-specific reporting

Life sciences study design

All studies must disclose on these points even when the disclosure is negative.

Sample size	*Describe how sample size was determined, detailing any statistical methods used to predetermine sample size OR if no sample-size calculation was performed, describe how sample sizes were chosen and provide a rationale for why these sample sizes are sufficient.*
Data exclusions	*Describe any data exclusions. If no data were excluded from the analyses, state so OR if data were excluded, describe the exclusions and the rationale behind them, indicating whether exclusion criteria were pre-established.*
Replication	*Describe the measures taken to verify the reproducibility of the experimental findings. If all attempts at replication were successful, confirm this OR if there are any findings that were not replicated or cannot be reproduced, note this and describe why.*
Randomization	*Describe how samples/organisms/participants were allocated into experimental groups. If allocation was not random, describe how covariates were controlled OR if this is not relevant to your study, explain why.*
Blinding	*Describe whether the investigators were blinded to group allocation during data collection and/or analysis. If blinding was not possible, describe why OR explain why blinding was not relevant to your study.*

Behavioural & social sciences study design

All studies must disclose on these points even when the disclosure is negative.

Study description	This survey-based quantitative study aims at investigating the labeling effects for the Taiwanese public between the terms "climate change" and "climate crisis."
Research sample	Our data set contained 1,892 individuals who were Taiwanese citizens and were at least 20 years-old. Among them, 934 (49.4%) participants answered questions that used the ‘climate change’ label, and 958 participants (50.6%) answered questions that used the ‘climate crisis’ label. The two samples did not differ from each other in terms of gender, educational attainment, or regional distribution. The two samples also did not differ from Taiwanese population in terms of gender and regional distribution, but did significantly differ from the Taiwanese population in terms of age. Compared with the Taiwanese population, both samples contained more older adults but fewer younger adults. As a result, out data set was representative of Taiwanese population in terms of gender and regional distribution, but was not representative of Taiwanese population in terms of age. This study targets adults population as they are important stakeholders to the climate policies in Taiwan. The reason we opted for a stratified sampling method is that this study wanted to include participants representative of all cities and counties of Taiwan.
Sampling strategy	This was a landline-based survey. We used a stratified sampling method. Survey samples were collected from the 22 cities and counties in Taiwan, with the number of participants surveyed proportionate to the number of population in a given city or county to the entire Taiwanese population. The research company called 18,524 numbers, and a valid response rate of 10-20% was expected. This resulted in a total sample size of 1,892. The sample size was limited by the research budget, but overall 1,892 respondents were considered sufficient to conduct the t-tests.
Data collection	The Computer Assisted Telephone Interview (CATI) system was used. The survey was performed by the Global View Survey Research Center, a Taiwanese research company. Possible participants were contacted via landline telephones. If the participants agreed to participate in this study, and they also provided verbal consent to participate in this study, then the trained interviewers read the scripts on the computer screen and inputted the participants' answers directly into the computers. The interviewers knew that there were two labels, but the respondents were unaware of this. At the start of the interview, the two labels were randomly chosen for each respondent. The researcher/researcher was not blinded to the experimental conditions.
Timing	The telephone survey was conducted between November 7, 2019 and November 18, 2019.
Data exclusions	For each statistical analysis, participants who did not answer the survey questions or stated that they did not know the answers were excluded from the analysis. We reported sample sizes for each of the analysis. As t-tests were conducted for 130 times for this study, and each has different sample sizes, please refer to Table 1, 2, 3, and 4 in the supplementary file for the respective sample sizes of each analysis.
Non-participation	We called 18,524 telephone numbers. Among them, 9,761 of the numbers (52.69%) were unsolved units (e.g., no one answered the phone, or it was a fax number). We terminated 3,760 of the numbers (20.3%) for reasons such as non-residential phone numbers, unqualified participants, or language barriers. About 17% of the calls (3,111 numbers, 16.79%) refused to participate in this study, stating reasons like not being interested in the topics, too many questions, or they simply refused to participate in any survey. We kept 1,892 samples (10.21%), which are the participants who finished all the questions, even if they did not answer every question or stated that they did not know how to answer the question.
Randomization	Participants were randomly chosen to answer questions that used either the "climate change" label or "climate crisis" label by the CATI system.

Ecological, evolutionary & environmental sciences study design

All studies must disclose on these points even when the disclosure is negative.

Study description	*Briefly describe the study. For quantitative data include treatment factors and interactions, design structure (e.g. factorial, nested, hierarchical), nature and number of experimental units and replicates.*
Research sample	*Describe the research sample (e.g. a group of tagged Passer domesticus, all Stenocereus thurberi within Organ Pipe Cactus National Monument), and provide a rationale for the sample choice. When relevant, describe the organism taxa, source, sex, age range and any manipulations. State what population the sample is meant to represent when applicable. For studies involving existing datasets, describe the data and its source.*
Sampling strategy	*Note the sampling procedure. Describe the statistical methods that were used to predetermine sample size OR if no sample-size calculation was performed, describe how sample sizes were chosen and provide a rationale for why these sample sizes are sufficient.*
Data collection	*Describe the data collection procedure, including who recorded the data and how.*
Timing and spatial scale	*Indicate the start and stop dates of data collection, noting the frequency and periodicity of sampling and providing a rationale for these choices. If there is a gap between collection periods, state the dates for each sample cohort. Specify the spatial scale from which the data are taken*
Data exclusions	*If no data were excluded from the analyses, state so OR if data were excluded, describe the exclusions and the rationale behind them, indicating whether exclusion criteria were pre-established.*
Reproducibility	*Describe the measures taken to verify the reproducibility of experimental findings. For each experiment, note whether any attempts to repeat the experiment failed OR state that all attempts to repeat the experiment were successful.*
Randomization	*Describe how samples/organisms/participants were allocated into groups. If allocation was not random, describe how covariates were controlled. If this is not relevant to your study, explain why.*
Blinding	*Describe the extent of blinding used during data acquisition and analysis. If blinding was not possible, describe why OR explain why blinding was not relevant to your study.*

Did the study involve field work? ☐ Yes ☐ No

Field work, collection and transport

Field conditions	*Describe the study conditions for field work, providing relevant parameters (e.g. temperature, rainfall).*
Location	*State the location of the sampling or experiment, providing relevant parameters (e.g. latitude and longitude, elevation, water depth).*
Access & import/export	*Describe the efforts you have made to access habitats and to collect and import/export your samples in a responsible manner and in compliance with local, national and international laws, noting any permits that were obtained (give the name of the issuing authority, the date of issue, and any identifying information).*
Disturbance	*Describe any disturbance caused by the study and how it was minimized.*

Reporting for specific materials, systems and methods

We require information from authors about some types of materials, experimental systems and methods used in many studies. Here, indicate whether each material, system or method listed is relevant to your study. If you are not sure if a list item applies to your research, read the appropriate section before selecting a response.

Materials & experimental systems

n/a	Involved in the study	
☒	☐	Antibodies
☒	☐	Eukaryotic cell lines
☒	☐	Palaeontology and archaeology
☒	☐	Animals and other organisms
☐	☒	Human research participants
☒	☐	Clinical data
☒	☐	Dual use research of concern

Methods

n/a	Involved in the study	
☒	☐	ChIP-seq
☒	☐	Flow cytometry
☒	☐	MRI-based neuroimaging

Human research participants

Policy information about studies involving human research participants

Population characteristics	See above.
Recruitment	See above.
Ethics oversight	Research Ethics Committee, National Taiwan Normal University.

Note that full information on the approval of the study protocol must also be provided in the manuscript.
